# The Pioneers of Nephrology **–** Professor Gabriel Richet: “I will maintain”

**DOI:** 10.1186/s12882-018-0862-0

**Published:** 2018-03-13

**Authors:** Giorgina Barbara Piccoli, Gilberto Richiero, Bernard G. Jaar

**Affiliations:** 1 Dipartimento di Scienze Cliniche e Biologiche, Università di Torino, Turin, Italy; 2 Nephrologie, Centre Hospitalier du Mans, 198 Avenue Roubillard, 72000 Le Mans, France; 3 via Susa 47 Chiusa san Michele 10050, Torino, Italy; 4 Department of Medicine, Johns Hopkins School of Medicine, Baltimore, MD USA; 5 Welch Center for Prevention, Epidemiology and Clinical Research, Johns Hopkins University, Baltimore, MD USA; 6 Department of Epidemiology, Johns Hopkins Bloomberg School of Public Health, Baltimore, MD USA; 7 Nephrology Center of Maryland Baltimore, Baltimore, MD USA

## Abstract

Gabriel Richet was one of the great pioneers of European Nephrology. After a pivotal period of work with Jean Hamburger, whom we owe the name of our discipline, Nephrology, he contributed to all aspects of this specialty and was, in particular, a forerunner in dialysis and in the study of interstitial nephropathies.

In this passionate and lucid interview, recorded in Paris in 2010, he describes himself as a “lucky man”, able to transform folly in happiness. He does not describe himself as an intellectual, but as a warrior, and closes a detailed history of the early days of European Nephrology with a strong statement of the moral stature a physician should have: he underlines, in line with his strong personality, that a physician is a man able to decide, to give orders and to assume their consequences. However, science and care of human beings cannot exist without a heart. “A doctor is someone who decides; when he writes a prescription, this means he prescribes and takes responsibility. Is it possible to give a prescription and decide regardless of compassion?”. In his interview, he commented that this last statement is probably not uniformly agreed, but that he’ll always defend it, adds freedom as a moral value that a physician should proudly defend: “Unfortunately I know that many do not share my idea, but that’s life... I am like the Queen of Holland, whose motto is: I will maintain!”.

## Introduction

*The series dedicated to the pioneers of the European Nephrology opens with the words of the pioneer of the pioneers, Gabriel Richet, interviewed in Paris in 2010.*

**Fig. 1:**
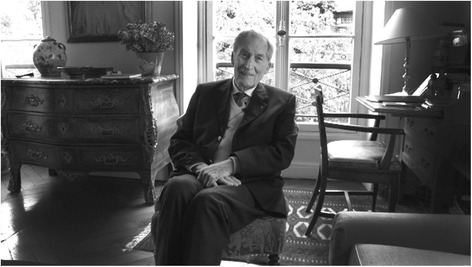
Gabriel Richet in his home in Paris in 2010. Photos by Gilberto Richiero

*Since no written English translation can account for the elegance of his irony or the intensity of his blue eyes, we invite you to listen to the full interview, of which we have selected sections that, in our opinion, better convey the heroic character of the lucky, sometimes a wild man, as he describes himself, in this passionate interview, reconstructing through enlightening anecdotes his personal history along with the history of Nephrology* [1] *(*Fig. 1*).*

## Professor Gabriel Richet: An interview

I’m a man who always had a lot of luck. I did a lot of silly things, and generally, their results have been quite good. That’s about it. I was very bold during the war in 39–40 and in 44–45 and also the time in between, but this was a bit different. I was injured, a bullet entered the top of my right thigh and was found against the left femoral artery, 60 cm of stitches and 60 days later, I went back to the front line. That is to say that I have a rather adventurous character.

Medicine? I don’t know why but I was a senior in high school; I was 16, I was bored during the correction of a Latin translation, I decided that I would study medicine, a Tuesday morning in February 1933. Of course, there was the weight of my name, my grandfather who had been awarded the Nobel Prize for Medicine for having discovered anaphylaxis ... he had twenty grandchildren, and I’m the only one to have studied medicine. My father and my mother, they were both physicians, my father was a professor at La Pitié hospital ...

This is the past onerous burden that made things easier for me, but which weighed heavily on my behaviour.

... There is nothing to do, yet you can do everything, try everything ... You can’t get rid of your ancestors. My father and mother were both doctors, they had six children and I’m the only one to have studied medicine. I think since my birth it was written; it could only be that.

Nephrology? I engaged myself when I was a resident of Pasteur Vallery-Radot, at Broussais hospital. I was not too happy there, and I enthusiastically followed Jean Hamburger in 1951, for a specialty that did not exist yet. Why do you want to have a specialty when there is no treatment?

Clinical nephrology was born, and well-born, when there were mavericks who invested in the treatment of acute uremia, but they made mistakes; they put all their efforts in chronic uremia where there was no hope.

Look at the first work of the Dutch Kolff which appeared in French in the “Presse Medicale” in April 1944; he put in his dialysis solution just water and sodium chloride. Potassium?... Did not know...! Acid-base? Did not know...! But he and Alwall in Sweden built the first dialyzers without taking into account the initial electrolyte content of the blood which was perfectly known for a long while. This mistake made us rediscover the notion of the *milieu interieur* (internal environment) of Claude Bernard and other authors. Henri Roger, Dean of the School of Medicine in Paris in 1925, used to say “Medicine, in particular, is a science that progresses by correcting mistakes more than by great discoveries.”

Medicine is made to cure diseases, so if you find something that you can’t use for healing, it could be very interesting, but you must go to the Faculty of Literature of Paris. But if you want to heal, you have to know the biological basics to conduct an intelligent therapy.

When we arrived with Jean Hamburger at Necker Hospital in January 1951, we didn’t know, and sometimes we didn’t want to know that urea and other nitrogenous waste products were not a direct cause of death, but the cause of death was the retention of potassium very nicely demonstrated by the work of Feltz and Ritter in 1881. You’ve probably never heard of these two people; you’ve probably never read their 300-page book ... but I must continue my education and yours at the same time!

And why has potassium been discovered? Because there were portable electrocardiographs… Suddenly, after the war, there were portable electrocardiogram machines with direct printing at the patients’ bedside. Once the peaked T wave of hyperkalemia was recognized, the treatment of AKI was transformed.

So this is to say that, first, it’s necessary to be specialized and secondly, it’s necessary to have a very open mind. How do you keep an open mind? Well, it’s extremely simple, it’s by examining your patients and with the idea of looking relentlessly for surprising facts.

The right to astonish oneself, the duty to astonish oneself, to find an anomaly in the reasoning process, a reasoning accepted by everyone else … by thinking outside of the box ...

I think this idea is still present in our specialty, and when our specialty evolves, we must have the courage, which I did not have, to abandon all your knowledge, in order to solve the unresolved issues …. One has to make the right choice at the right time to resolve the problem.

I remember one of my bosses whose name I will not mention ... When I started to practice in ‘45, returning from the war, we were all practicing internal medicine, and there were older persons with back pain who were given large doses of vitamin D, and it worked. My boss told me: “I administered it, but I do not know how it works, but empirically, it works” and then we learned that these patients had uremic osteopathy with all that it entails, and the kidney was an organ that transformed vitamin D ...

As you know, the whole Paris school focused on acute uremia, once we understood that it interposed itself between the severe infection and death, but it could be resolved like some rare cases of toxic uremia.

The experience with acute uremia was encouraging ... patients could be cured and not dying because we could not keep them alive for two weeks, because we could not control their biochemical disorders. And that is something that is very important because it changed the way of thinking, broadening it beyond nephrology, as we shall see later.

The first attempt which was made in France in 1947 was successful; it was the exchange transfusion, seven or eight liters of blood, and this was due to the work of Paul Milliez in the service of Pasteur Vallery Radot. He succeeded him one decade later. This method did not last because there were difficulties in obtaining enough liters of blood, we only knew the Rh factor apart from other major blood groups … but also its efficacy was mediocre.

The second attempt was made at the Necker Hospital. It was the intestinal infusion by a naso-jejunal probe with evacuation by natural routes.

There was a patient whom I remember. She came to Necker on June 6th, 1951. She was a mother of four children, her last child was born a few days earlier, and she developed a severe pelvic infection that was successfully treated with penicillin, but she remained anuric. But then we were doubly lucky: she was a robust peasant, I would say like a Verdun soldier or the equivalent (…). We gave her an infusion probe. After 24 h, she couldn’t stand the probe any longer and she ripped it out. Her husband came to see her at the end of the morning, and Professor Hamburger told him: “We’re sorry, but your wife just ripped out her probe and it was her only chance of survival”. Her husband answered: “Fine”, went to see his wife who was nearby, and said (…) “You have cost me a lot of money in ambulance and transport fees to come to Paris from Loir-et-Cher, and then you rip out your probe, what am I going to do with our four kids?” (…). After this, she kept her infusion probe for 11 days, and she recovered.

I can tell you that this case study, which we published in the Société Médicale des Hôpitaux, taught us all about spontaneously reversible acute kidney injury...

Not only this technic kept her alive, but it kept alive a mother for her children, a wife for her husband, whose strength she admired. She served as a reference for future cases.

Then there was peritoneal dialysis. The first peritoneal dialysis, I don’t know where they happened, but in France, they began in ‘47 with Pierre Tanret at Hotel Dieu hospital who tried peritoneal dialysis, it was an amazing DIY [*Do It Yourself*]...

They were very complicated treatments: intestinal dialysis, peritoneal dialysis, and artificial kidney. We went from biology to very simple physico-chemical laws: it was called dialysis, described in 1825 by Dutrochet. It was famous, but it was forgotten...

We forgot that there were people who worked before the end of the Second World War.

I’ll tell you what happened to me. In the United States, the artificial kidney has benefited from the work of a person who had done a great job, Merrill in Boston, who in 1952 published an excellent article in “Medicine” about it. But everywhere else, nobody cared about dialysis, everyone, or almost everyone, thought that conservative management was the only one that could work ….

Well, I was in New York in ‘54, and a senior told me: “Why the hell did you go to Boston to learn the handling of the Kolff Merrill artificial kidney, while in New York we don't need it?...” They told me that in 1954, but why? Because patients were treated in internal medicine, where they saw a case of anuria every six months, while Merrill used to accept all the anuric patients, sent to him by plane, from all over the United States.

At the time of the war in Algeria, with injured people that often were highly infected, at Necker, we accepted patients coming from all over France, from Algeria and even from Chad, as the French Military Health Services and Social Security paid their transport and treatment. It’s interesting to see how social policy is involved in the implementation of treatments. Through French social policy, in Paris, then quickly in Lyon and Toulouse, and after everywhere in France, we have gained experience with these patients. I will not discuss further but to say that, three years later in 1957, I went to South America to install the first artificial kidney in Sao Paulo, because there was a South American student who spent some time with us at Necker.

And you will find the same problem during development of renal biopsy. There comes a time, when you want to get ahead and get right down to applying a new observation, a new treatment, where patients must be grouped together so that you know immediately how to get and interpret the results.

At that time, the artificial kidney, the treatment of acute kidney injury by dialysis, was the basis of the principle of intensive care. I’ll show you a book on intensive care that I published with Hamburger and Crosnier, which we wrote in September ‘52, which was printed in March ‘54 by Flammarion; it was the first book about intensive care, the gateway to the intensive care unit (ICU).

And so, that’s why we started with this. After I’ll tell you why we abandoned it.

Why did we start with this? Because returning from the summer holidays in 1952, we sat down and reviewed what had happened in the previous months. We did not yet have the artificial kidney, we had in mind that there were other diseases than renal diseases that were potentially life-threatening, but possibly reversible.

Hamburger and I were driving to visit a patient at the American Hospital of Paris, and we were crossing the Bois de Boulogne; it was the usual route, and during this trip, we did not need to pay attention to the .... Hamburger said, “Well, we may organize a day or two dedicated to intensive care, but should we continue following this path or give up?”.

He asked me the question, and I said “Why not? Let’s continue.” We talked again a few days later and he said: “We should stop, because you should never have two goals in life,” and I told him that when I was in the French commando unit, an older lieutenant told me: “If you have two goals, you’ll reach none and there is a high risk of being killed. No second goal.”.

(…)

Now let’s finish with the case of Marius Renard.

Marius Renard, his name tells you nothing. But, on December 18th, 1952, Marius Renard, who lived in a small village 60 km North of Paris, near Beauvais, was an apprentice roofer who fell from a roof three stories high on his right kidney, which ruptured, so the surgeon removed it, because he had huge hematuria and became anuric.

What the surgeon did not know was that this poor kid had only one functioning kidney. He was taken to Paris, Necker; the urologists referred him to us because they did not know what to do. His mother proposed to donate one of her kidneys because she had seen a movie where there was supposedly a kidney transplant.

You must take into account all the social life factors as well as all new developments in medicine.

The mother had almost all blood groups, and all subgroups identical to those of her son and a kidney transplant was carried out on December 25th, 1952 at 9 pm at the Necker Hospital in Paris. A few days later, the news spread in the press, we’ll see later; the kidney worked, but the glomerular filtration was never very good.

The urea clearance was 25 ml and dropped dramatically to 18 ml 20 days after transplantation, and then it stopped suddenly. We re-operated to find out if there was not a surgical complication, but there was nothing to do, and death came.

And with this story we had three conclusions: first, there was the HLA system that we did not know about yet, Dausset was just beginning his work. There were small differences in subgroups; it was intellectually the same as a crash incompatibility transfusion.

Secondly, there were a number of small signs: the kidney became a little bigger, blood pressure measured every hour rose 5 mm during the last two days, from the 19th to 20th day. There was another small sign; there was a trace of albumin in the urine, which did not exist before. Therefore, there were all the clinical signs, which lead to the diagnosis of rejection, which we described at this time.

Third, there was a global social impact. Marius died; at his funeral, in his village, 50 m of street were blocked by flowers coming from all over the world ... All over the world people sent telegrams. Some said they were ready to donate a kidney to attempt a second transplant for Marius. As a consequence, you know, the world public opinion told Medicine: “Find a solution for kidney transplants.”.

And it is always thinking about well-observed unique cases, which makes us progress in medicine.

The institution “Assistance Publique-Hôpitaux de Paris” immediately considered the Nephrology department of Necker as the place where it was necessary to do renovation work, and we went from 40 to 100 square meters, and further. And seven years later, in ‘59, there was the Merrill transplantation technique, followed by the Hamburger’s transplantation, but at that time I was just a witness and no longer an actor.

That’s what I can tell you about how amazing this story was ... it’s necessary to have an educated mind, but with an education that has not demolished the thinking process. If I only told you that, and if you let yourselves be seduced by the old man that I am, do read “The Thibaults” which has 3000 pages ...

(…)

Choosing medicine? That doesn’t mean specifically nephrology, I would tell a student: “Choose a branch that you love; to love it, you must know it, therefore, you must have measured its past and its future potentials, you must simultaneously conduct a creative scientific work, but animated, nourished by a medical culture”.

We are doctors to heal, period.

If we do not have a healing goal, we only make beautiful literature, but I leave this to others. What is stupid, profoundly stupid in our medical profession, is to believe that because you are protected by a powerful university, you become important. In this case, that kind of importance is nonsense. You can write it; I do not mind.

Does poetry exist in our medical profession? Well, I do not know; (…) because in poetry you need to read verses, or to express your thought with delicacy and strength ...

But I know that without a heart, medicine does not exist.

Computer medicine? it’s not possible. It’s just like Justice. Why are there still judges and not just computers? It is because there are things you can’t put in a computer.

A doctor is someone who decides; when he writes, he writes a prescription, this means he prescribes and takes responsibility.

Is it possible to give a prescription and decide regardless of compassion? Unfortunately, I know that many do not share my idea, but that’s life.....

I am like the Queen of Holland, whose motto is: “I will maintain!”

## Professor Gabriel Richet: A synopsis of his life and achievements

Gabriel Richet was born in 1916 in Paris, from a brilliant dynasty of physicians: his father, Charles Richet junior, was a specialist in human nutrition, Professor at the Faculty of Medicine in Paris. His mother, Marthe Trélat was one of the first women who became *interne* (resident) in the hospitals of Paris. His grandfather, Charles Richet, was a Nobel laureate in 1913, for the discovery of anaphylaxis.

During the Second World War, he participated in the Campaign of France in 1940 and in the combats in the Vosges Mountains in 1945 where he was wounded. He was a decorated World War Two veteran and received the award of “*Chevalier de la Légion d’Honneur”* by General de Gaulle in Karlsruhe in April 1945.

From 1950 to 1960 he was with Jean Hamburger, the founder of French and international Nephrology, at Necker Hospital in Paris; both are considered belonging to the post-war rebuilders of the French academic medicine.

After having spent three months in Boston in the department of nephrology directed by John Merrill, he realized the first hemodialysis in France and was involved in the first allogenic transplantation that was not immediately rejected. Together with Jean Hamburger and Jean Crosnier, he developed the concept of renal intensive care aimed at correcting disorders of the major fluid electrolyte, acid-base, and other metabolic functions, thereby markedly improving the prognosis of patients with acute kidney injury.

In 1961, he founded the nephrology service at Tenon Hospital and attracted in his clinical department as well as in his research laboratory, many young collaborators, and scientists. Among his pivotal contributions, was the characterization, with Jacqueline Hagège and Manfred Gabe, of the dark cells (intercalated cells) of the collecting duct, which play a key role in acid-base regulation. He was also, with his group, a founder of translational medicine long before this name was coined, bringing to the lab unusual cases and conversely translating into clinical applications the results obtained in experimental models.

He was a founding member of the International Society of Nephrology (ISN) in 1960, and President of the ISN between 1981 and 1984.

After his retirement in 1985, he remained an active member of the French Academy of Medicine and devoted much of his time to the history of medicine and, particularly, of nephrology.

He received numerous honors and awards, including honoris causa degrees, and the prestigious Jean Hamburger award of the International Society of Nephrology in 1993.

**Fig. 2:**
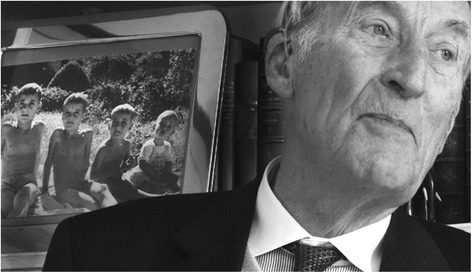
Gabriel Richet in his home in Paris in 2010. Photos by Gilberto Richiero

**Fig. 3:**
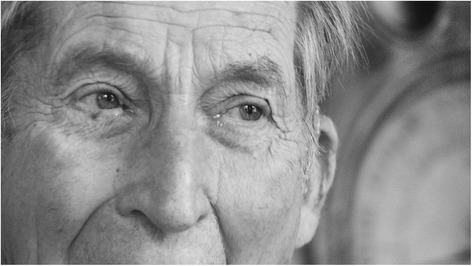
Gabriel Richet in his home in Paris in 2010. Photos by Gilberto Richiero

**Fig. 4:**
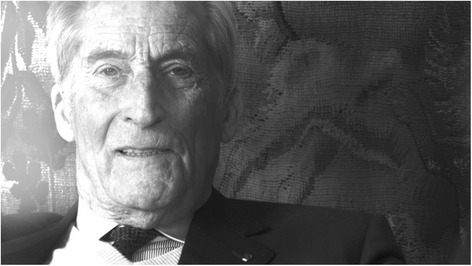
Gabriel Richet in his home in Paris in 2010. Photos by Gilberto Richiero

In 2012, he was appointed Grand Officier de la Légion d’honneur of France, one of the highest honors of the French Republic (Figs. 2, 3 and 4).
